# The Implantation of Teeth

**Published:** 1889-02

**Authors:** G. L. Curtis

**Affiliations:** Syracuse, N. Y.


### THE IMPLANTATION OF TEETH.*

BY G. L. CURTIS, M.D., D.D.S., SYRACUSE, N. Y.

As the scientific aspect of this subject has so recently been considered, your attention to-day will be directed to the practical side of this comparatively new field in dental surgery. Few subjects in our profession have been more generally and more thoroughly deliberated over than this. While at present many are not sanguine that this method of restoring the lost organ will be ultimately successful, it must be remembered that our work is largely experimental. We are meeting the failures as they appear, and endeavoring to overcome them. Farther than this, we are devising new ways whereby natural teeth can be permanently fixed in the jaw. If perfection has not already been attained, it will, we are confident, be reached in due season. Where there is a demand, the way to supply it generally exists. If the world-renowned American dentist, with his intelligence, energy and ambition cannot accomplish the task before him, then he must "call upon the angels for inspiration."

For three years implantation, which already may be pronounced a justifiable and proper practice, has been prosecuted with the most gratifying results. I take pleasure in reporting to you that teeth thus embedded in the jaw two years ago remain intact, and are as useful, and to all appearances as healthy, as any others in the arch.

We are experimenting in this work entirely from a scientific point of view, and under the most favorable conditions. Hence our failures, when they do occur, enable us to consider the causes, and lead us to the discovery of better modes of operating. Is it strange that failures have to be recorded? Hundreds of teeth have been implanted; many of them under circumstances most unpromising, and by operators who for the first time performed a surgical operation greater than the extraction of a molar, and whose knowledge of surgery and pathology never extended beyond the carious tooth. Name the surgeon who, after years of experience, is prepared to affirm that he knows that his operations will be successes, and you have pronounced the name of a knave.

I have little sympathy with those who condemn every venture in scientific work, and who are ready to ridicule and censure all whose methods differ from their own. By our failures we learn to

---

* Read before the New Jersey State Society, at its Eighteenth Annual Session, held at Asbury Park, July 19, 1888.

overcome the obstacles in the path to success. Were we afraid to explore the unknown, or were we restrained by rigid dogmas, advancement would be impossible.

In performing implantation, the fully developed and healthy tooth is best adapted for the work. The objection to teeth of the youth, such as are removed in correcting irregularities, is that the root is undeveloped, while those from the patient of advanced years are prone to disease or are already affected. When teeth intended for implantation have become dry, they should be allowed to remain for a few days in a weak solution of bichloride of mercury—about one to five thousand. By this means the original color will be restored, and the chances of fracturing them while cleansing the canal are lessened. When the alveolar wall is almost or quite absorbed, only the longest teeth can be used, and the articulation maintained. In the last named case, roots with artificial crowns attached will supply the demand. These can also be employed when the palatal root of a molar is used to supply the loss of a lateral incisor.

Too much stress cannot be laid on the importance of selecting suitable teeth and of choosing the mode of operation demanded by the case under treatment. When the alveolar ridge is full and well developed, the condition is the most favorable. When it is atrophied, short teeth, or those with long crowns should be selected. This permits a complete embedding of the cementum, and thus the chances from exposure to septic influences are reduced. As living substances readily unite, we argue that freshly extracted teeth can best be employed. Their dentinal fibres and cementum are prepared to continue life, while experiments show that dried and shriveled teeth have lost the capacity of taking on life again. In the dry tooth checking has perhaps resulted, thus diminishing the chances of durability.

In preparing the tooth, all foreign and unhealthy substances should be removed from it. I have seen teeth implanted with fragments of dead pericemental membrane, salivary calculus and even parts of abscess sacks clinging to the cementum. It is hardly necessary to add that in such cases the results will likely be disastrous. The removal of the pulp and the filling of the canal can best be accomplished through an opening made in the palatal or masticating surface. Gutta-percha is probably the best filling substance. Opening through the root wounds the cementum, and increases the chances of absorption. Indeed, the wound caused by the use of the forceps during the process of extraction may be the beginning of the breaking down of the cementum, as has been shown in the brief history of implantation.

In selecting patients for this operation, the scrofulous, those in the primary or secondary stage of syphilis, those affected by any wasting disease, and those whose system is overtaxed, or who are in the period of gestation, should be declined. It is clear that such persons are not in a condition of body which will result in quick or satisfactory healing.

In forming the socket, great care should be observed to obtain an accurate adjustment of the root. If this is done, nature in her work of reproducing the bone tissue is less taxed, while the likelihood of absorption is lessened. When the alveolar ridge is entirely wanting, and the other conditions are favorable, cut through into the substance of the bone and insert the tooth. This can be done with the same assurances of firm attachment as in the other operations. The danger of penetrating the antrum should not be overlooked; yet in cases where this has occurred no ill results have been reported.

When all is ready for the insertion of the tooth, place it for a few minutes in the sterilizing solution, the temperature being slightly above that of the body. This prevents the coagulation of the blood from the wounded bone. At the same time syringe the socket with a similar solution, and remove all clotted blood and fragments of bone. This done, press the tooth firmly in place, and anchor it to an adjoining tooth. The firm retention of the implanted organ in its new position until the process of healing is complete, by means of a staple or clasp, is essential. This fact was thoroughly demonstrated in the papers read at the last annual meeting of the First District Dental Society of New York.

If undue inflammation occurs, meet it as in the case of an acute abscess, the cold compress being one of the most effective agents.

In the cases of failures reported, there appeared to be two methods of the absorption of the root. In the first, bacteria play an important part; and, in the second, the bone cells, in penetrating the cementum and dentine, apparently know no limit, and the root is disposed of as in sponge grafting. Finally the crown, receiving support only by its attachment to the periosteum, requires but a slight pressure to be displaced.

Cannot the success of implantation in a measure at least be attributed to natural organization or condition, that is, to the temperament? In view of the remarkable differences that exist among individuals, in consequence of the variety of the relations and proportions of the constituent parts of the body, does it not seem reasonable that teeth implanted with due regard to these conditions will be more likely to be permanently retained? May not this also account for the promptness of the healing process, and the readiness with which some teeth assume the color of their neighbors? Thus, teeth belonging to the bilious temperament, when placed under like influences, assume a normal condition and remain in it more readily than when these relations are antagonistic. The color, however, might be accounted for by the pigment peculiar to the possession of the tooth, as in all cases we do not find a perfect blending of shades. Again, this may be attributed to the septic influence of the devitalized dentinal fibres remaining in the tooth. This point is open for your consideration.

The introduction of implantation has already proved a great benefit to our profession, and its beneficent work is but begun. The investigators who have taken up this work deserve commendation; but special praise is due to him who first conceived the idea, and first put it in execution. The work already done, even if it shall prove to have been largely misdirected, will surely lead to a method whereby teeth can be permanently attached in the edentulous jaw. Thus, if bone cells have to do with the destruction of the roots as in sponge grafting, cannot the root be partly surrounded with an impervious substance which will be retained in the jaw, and which will hold the crown when the root is thrown off?

I am glad to see so much interest manifested in this subject here, and apparently in its favor. I have been surprised to find almost every one condemning the operation heretofore. It is true that a few failures have been reported, and they seem to have been selected with a view of destroying the confidene of the profession in the operation by men who are positively opposed to it; for what reason I do not know.

But the success which has already been reached under the present methods commends implantation to the scientific dentist, as well as to the crippled patient; for who is more maimed than he who has been the prey of the hungry knight of the forceps? For years the need of better substitutes than artificial dentures has been keenly felt, and have we not strong ground to hope that this need was answered when Dr. Younger introduced to the world this astonishing innovation in dental science?

